# Benzimidazole resistance in *Haemonchus contortus* in small ruminants: molecular mechanisms and diagnostic approaches

**DOI:** 10.1016/j.ijpddr.2026.100652

**Published:** 2026-06-09

**Authors:** Zofia Nowek, Marcin Mickiewicz, Alistair Antonopoulos, Adrian-Valentin Potărniche, Kinga Biernacka, Rareş-Constantin Bejenariu, Karolina Radziejewska, Aija Mālniece, Jarosław Kaba

**Affiliations:** a Division of Veterinary Epidemiology and Economics, Institute of Veterinary Medicine, Warsaw University of Life Sciences–SGGW, Warsaw, 02-776, Poland; b Kreavet, Kruibeke, 9150, Belgium; c Department of Infectious Diseases and Preventive Medicine, Law and Ethics, University of Agricultural Sciences and Veterinary Medicine, Cluj-Napoca, 400372, Romania; d Clinical Institute, Faculty of Veterinary Medicine, Latvia University of Life Sciences and Technologies, Jelgava, LV-3004, Latvia

**Keywords:** Gastrointestinal nematodes, *Haemonchus contortus*, Anthelmintic resistance, Benzimidazoles, Molecular diagnostics, Small ruminants

## Abstract

Benzimidazole resistance in *Haemonchus contortus* represents a widespread challenge in small ruminant production worldwide. Benzimidazoles remain one of the most commonly used drug classes, due to their broad spectrum of activity, affordability, and ease of administration. However, extensive and often inappropriate use of these compounds has led to the rapid emergence and global spread of resistance, particularly in *H. contortus*, a highly pathogenic and genetically diverse gastrointestinal nematode. Resistance to benzimidazoles is primarily associated with point mutations in the isotype-1 β-tubulin gene, most notably at codons 167 (F167Y), 198 (E198A), and 200 (F200Y), which result in modifications to the β-tubulin that prevent drug binding and compromise treatment efficacy. This review summarises the molecular mechanisms underlying benzimidazole resistance and provides an overview of currently available diagnostic approaches, including *in vivo* and *in vitro* assays, as well as molecular techniques such as real-time PCR, digital PCR, pyrosequencing, deep amplicon sequencing, and isothermal amplification methods. Despite regional variation in the distribution of resistance-associated polymorphisms, there is a consistent global predominance of the F200Y mutation. Finally, this review highlights the importance of integrating molecular surveillance with sustainable parasite control strategies to slow the further spread of benzimidazole resistance and preserve the long-term efficacy of available anthelmintics.

## Highlights

• Benzimidazole resistance is widespread in *H. contortus* populations.
• Key resistance SNPs in isotype-1 β-tubulin include F167Y, E198A and F200Y.
• Molecular assays enable sensitive detection of resistance-associated SNPs.
• F200Y remains the predominant SNP across global *H. contortus* populations.

## Graphical abstract

**Figure:**
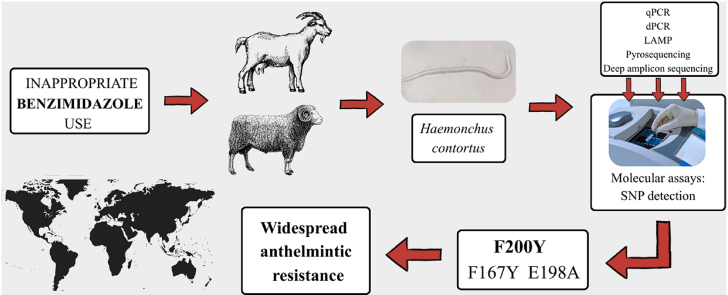

## 1 Background

Parasites pose a threat to both animal welfare and the economic viability of many ruminant farming systems worldwide. Gastrointestinal nematode (GIN) infections in ruminant livestock are of major importance and cause significant production losses (Byford et al., 1992; Charlier et al., 2014,^2020^; Henrioud, 2011). Most of these nematode species belong to the *Strongylida* order and the *Trichostrongyloidea* family. The most common genera that infect small and large ruminants are *Haemonchus*, *Cooperia, Ostertagia, Teladorsagia*, *Bunostomum, Trichostrongylus, Oesophagostomum, Chabertia, Nematodirus, Protostrongylus*, and *Trichuris*. They typically reside within the abomasum, small intestine, and, less frequently, the large intestine (do Amarante and Vendrame Amarante, 2016; Belina et al., 2017). In general, gastrointestinal nematodes suppress the host's appetite and foraging behaviour, and/or decrease the effectiveness of nutrient absorption (Coop and Kyriazakis, 2001). These infestations result in anaemia, diarrhoea, weakness, losses in meat, milk, and wool production, progressive body weight loss, wasting, and death (González et al., 2003; Smith and Sherman, 2009; Toscan et al., 2017).

In small ruminants, the GINs of most importance, both economically and for animal health and welfare, are *Haemonchus contortus, Teladorsagia* spp. and *Trichostrongylus* spp (Charlier et al., 2020). Most infections tend to be mixed, and it is often difficult to link a single parasite to the clinical symptoms (Smith and Sherman, 2009). The clinical picture produced by *Teladorsagia* spp. and *Trichostrongylus* spp. is similar, with the main symptoms including anorexia, diarrhoea, dehydration, intermandibular oedema, a pot-bellied appearance, a rough and dry haircoat, flaky skin, and weight loss (Craig, 2009; Smith and Sherman, 2009).

However, *H*. *contortus* is one of the most prevalent and invasive of these species (Emery et al., 2016; Goel et al., 2020; Iliev et al., 2017). Originating in sub-Saharan Africa, it was disseminated worldwide through livestock movement over the past centuries (Gilleard, 2013; Hoberg et al., 2001). Due to its tropical origin, the parasite's free-living larval stages are not well adapted to survive cold winters in temperate climates, and it persists mainly within the host during colder periods (Waller et al., 2004). A study conducted in Polish goat herds by Mickiewicz et al. (2021) exposed *H. contortus* as the predominant species among those examined. The sucking of blood by these parasites and the eruption of ulcerative lesions in the abomasal mucosa cause significant pathogenic damage, leading to digestive syndrome and anaemic disorders (Besier et al., 2016). Moreover, its high propensity to develop resistance to anthelminthic drugs makes treatment more difficult (Kotze and Prichard, 2016). Clinical signs of anaemia predominate in haemonchosis. Affected animals present pallor of the conjunctiva and mucous membranes, as well as intermandibular oedema (bottle jaw), caused by loss of plasma proteins (Dwight and Bowman, 2021). When haemonchosis is not complicated, constipation is more common than diarrhoea. Additionally, weight loss, anorexia and agalactia are common findings in prolonged illness (Besier et al., 2016; Iliev et al., 2017; Smith and Sherman, 2009).

## 2 Anthelmintic use and the emergence of resistance in gastrointestinal nematodes

Since the advent of broad-spectrum anthelmintics, control of gastrointestinal nematodes (GIN) in goats has historically relied on the frequent administration of three principal anthelmintic classes: benzimidazoles (BZs) e.g.: albendazole, fenbendazole, oxfendazole; macrocyclic lactones (MLs) e.g.: ivermectin, doramectin, eprinomectin, and imidazothiazoles (primarily levamisole, LEV)-throughout entire flocks (van Wyk, 2011). Routine use of these anthelmintics (often without prior assessment of parasitic burden) has contributed to the emergence of anthelmintic resistance (AR) in nematodes. This applies to various GIN species, which have become resistant to major drug classes (Falzon et al., 2014; Kotze and Prichard, 2016; Waller, 1994).

Resistance is defined as the ability of an organism to survive drug doses that would usually be lethal to others of the same species and stage. In gastrointestinal nematodes such as *H*. *contortus,* it is understood as a reduction in efficacy compared to when the drug was first introduced into field use (Kaplan et al., 2023; Kotze and Prichard, 2016; Prichard, 1994). Although new anthelmintic classes, such as amino-acetonitrile derivative monepantel and spiroindole compound derquantel, have been developed (Kaminsky et al., 2008, 2011), resistance to monepantel emerged as early as two years after its introduction (Sales and Love, 2016; Van den Brom et al., 2015). In Poland, the anthelmintics most frequently used in goat treatment are albendazole, fenbendazole, and eprinomectin, reflecting the observed AR to BZs and MLs (Mickiewicz et al., 2021).

## 3 Alternative control methods for GIN

In addition to chemotherapeutic treatment, methods for limiting the burden of these nematodes include grazing management, refugia and biological control (Charlier et al., 2024; Hodgkinson et al., 2019). Grazing management involves strategic rotation of pastures, mixed-species grazing, and targeted stocking density to break the nematode lifecycle and reduce pasture contamination. Changing grazing patterns and providing rest periods for pastures, can interrupt the lifecycle of nematodes, thereby decreasing their overall population (Domke et al., 2011; Githigia et al., 2001; Larsen, 2006; Mahieu and Aumont, 2009; Taylor et al., 1991; Thamsborg et al., 1999). Another important concept in sustainable parasite management is refugia, which refers to the portion of a parasite population that remains unexposed to treatment. In this context, untreated hosts or environmental stages of parasites help maintain drug-sensitive genotypes within the population, thereby reducing selection pressure for resistance. The refugia-based approach, first described by van Wyk (2001), involves treatment of only a proportion of animals rather than entire flocks. This leaves some parasites unexposed to drugs, which helps to slow the spread of AR (Hodgkinson et al., 2019; van Wyk, 2001). Biological control is defined as: “any activity of one species that reduces the adverse effect of another” (Alston, 2011). Certain plants, especially those high in tannins or flavonoids, have demonstrated anthelmintic effects against gastrointestinal nematodes in ruminants. These plants interfere with the lifecycle of nematodes or alter the host's immune response. Incorporating them into pastures or adding them to animal diets can enhance sustainable parasite management strategies in ruminant production systems (Athanasiadou et al., 2007; Hoste et al., 2012, 2015; Kwenti, 2017). Moreover, less-known biocontrol agents of GIN in grazing animals include nematophagous fungi, earthworms, dung beetles, predatory nematodes, and nematophagous mites (Szewc et al., 2021; Waller, 2006). Nevertheless, the wider adoption of biocontrol agents and high-tannin forages in parasite control programs will depend on their economic feasibility, particularly whether their antiparasitic benefits outweigh the costs associated with establishment, management, and variable efficacy under different production conditions (Hoste et al., 2012; Faria et al., 2025).

## 4 Benzimidazoles: mechanism of action and resistance in GIN

BZs stand out as one of the most frequently used anthelmintic drug classes, prized for their affordability, broad spectrum of activity, and straightforward administration. Within this group, notable examples include fenbendazole, oxfendazole, thiabendazole, and oxibendazole. However, albendazole and mebendazole hold particular prominence as widely recognised drugs, extensively employed in both human and veterinary medicine (Barrère et al., 2012; Boatin et al., 2012; Sangster et al., 1985; Swanson et al., 2012). BZs share the same mechanism of action, selectively binding to nematode β-tubulin inhibiting the polymerisation of α- and β-tubulin dimers into microtubules. This disrupts the formation of the cytoskeleton, the mitotic spindle, and intracellular transport. As a result, the parasite metabolism is compromised, ultimately leading to its death (Lacey, 1988). However, inappropriate and excessive use of these drugs has led to the development of AR (Wolstenholme et al., 2004).

The mechanism of resistance to BZs has been extensively studied over the past few decades. It is considered the most comprehensively understood from a molecular perspective, compared to other classes of anthelmintics (Kotze et al., 2014). Point-substituting mutations in the isotype-1 β-tubulin gene cause modifications to the β-tubulin structure via amino acid changes, which prevent BZs from binding (Lacey and Gill, 1994). The first single nucleotide polymorphism (SNP) linked to BZ resistance was discovered in codon 200, leading to the F200Y (TTC to TAC), which causes tyrosine to be expressed instead of phenylalanine (Kwa et al. 1994, 1995). Additionally, it has been observed that resistance is also linked to two amino acid changes in two other codons: F167Y (TTC to TAC) and E198A (GAA to GCA) (Ghisi et al., 2007; Prichard, 2001, Silvestre and Cabaret, 2002). The following three SNPs in the isotype-1 β-tubulin gene have been reported for various ruminant parasitic nematodes, including *H*. *contortus*, *Teladorsagia circumcincta*, *Cooperia oncophora*, and *Ostertagia ostertagi* (von Samson-Himmelstjerna et al., 2007). A range of additional SNPs have also been reported, in both isotype-1 and isotype-2 of β-tubulin in parasitic nematodes (Doyle et al., 2022; Francis et al., 2024; Martinez-Valladares et al., 2020), although the classical three SNPs in codons 167, 198, and 200 tend to predominate. Importantly, the inheritance pattern of BZ resistance is autosomal and exhibits semi-dominance. Consequently, resistance is expressed in a graded rather than a strictly binary manner: heterozygotes carrying one mutated and one non-mutated allele display lower resistance than homozygotes with two mutated alleles. At the same time, full susceptibility occurs only in homozygotes with two non-mutated alleles (Sangster et al., 1998).

## 5 Detection of anthelmintic resistance

Currently available methods for detecting drug resistance in gastrointestinal nematodes include *in vivo* methods, primarily the faecal egg reduction test (FECRT), *in vitro* methods, such as the egg hatch test (EHT) and larval development test (LDT), and molecular techniques, mainly PCR and pyrosequencing (Kotze and Prichard, 2016). Due to the mechanism of action of BZs, newer high-throughput *in vitro* technologies such as the automated larval migration assay (ALMA) (Blanchard et al., 2018; Harrington et al., 2023) and the WMicrotracker (Alberich et al., 2025) are not suitable for the detection of BZ resistance. Both *in vivo* and *in vitro* methods have their own limitations. FECRT is expensive and time-consuming, and there is significant diversity between the animals; furthermore, the pharmacokinetics of the drug in the host can lead to inaccurate results (Babják et al., 2018). In addition, the FECRT does not directly measure resistance, but rather treatment failure, which can be influenced by a wide range of additional factors beyond resistance (Morgan et al., 2022). Recently, new guidelines were released for the FECRT, which is expected to address some of these limitations (Kaplan et al., 2023). Conversely, the sensitivity of EHT and LDT can often be insufficient. This is the case when less than 25% of the parasite population is resistant (Martin et al., 1989). Moreover, daily fluctuation in the effective dose (ED_50_) and the lethal concentration (LC_50_) during the pre-patent period can also influence the sensitivity of both tests (Borgsteede and Couwenberg, 1987; Hubert and Kerboeuf, 1984). On the other hand, molecular techniques have the advantage of enabling the quantification of allele frequencies in DNA extracted from field samples. If a pooled stool sample is used, the results accurately reflect the herd's current condition (Ramünke et al., 2016). Nowadays, screening SNPs is considered the most sensitive and efficient method for examining field samples for BZ resistance. Molecular tests include conventional PCR, real-time PCR, digital PCR (dPCR), pyrosequencing (Esteban-Ballesteros et al., 2017; Barrère et al., 2013b; Höglund et al., 2009; Ramünke et al., 2016), deep-amplicon sequencing (Avramenko et al., 2015, 2019; Francis et al., 2024), and isothermal amplification technologies such as loop-mediated isothermal amplification (LAMP) (Costa-Junior et al., 2022).

### 5.1 Conventional PCR and real-time PCR

Conventional PCR uses primers that target known genetic markers associated with BZ resistance in parasitic nematodes. After amplification, the PCR products are visualised using gel electrophoresis, allowing for the detection of specific DNA fragments indicative of resistance. Several techniques of this kind have been reported to detect resistant individual trichostrongylids (Elard et al., 1999; Silvestre and Humbert, 2000). A further application of this technology is the restriction fragment length polymorphism (RFLP) PCR, which uses restriction enzymes to selectively fragment PCR products based on the presence of absence of the SNP of interest, and has also been successfully demonstrated for BZ resistance detection (Tiwari et al., 2006). A significant limitation of these methods stems from their reliance on agarose gel electrophoresis for assessing the allele status of individual nematodes. Consequently, conducting these reactions on an adequate number of specimens to derive meaningful allele frequency data becomes a time-consuming and labour-intensive endeavour. Real-time PCR (qPCR) allows for the simultaneous amplification and quantification of DNA associated with BZ resistant alleles. Fluorescent probes or DNA-binding dyes are used to monitor amplification in real-time, enabling the accurate measurement of DNA concentration (von Samson-Himmelstjerna et al., 2003; Walsh et al., 2007). Nevertheless, the application of qPCR for SNP detection is constrained by its targeted nature, as it can only detect previously characterised mutations and may be affected by low-abundance alleles or polymorphisms within primer- and probe-binding sites, which can compromise the accuracy of allele discrimination and frequency estimation (von Samson-Himmelstjerna et al., 2003; Walsh et al., 2007).

### 5.2 Digital PCR systems

Digital PCR (dPCR) provides absolute quantification of nucleic acids, serving as an alternative to standard quantitative PCR (qPCR). In dPCR, the sample is divided into multiple independent PCR reactions, with each partition containing either none or several copies of the target sequence. After amplification, each partition is evaluated for the presence of the target based on fluorescence: positive partitions indicate successful amplification, while negative ones do not. The concentration of the target sequence in the original sample can be determined with statistically specified accuracy by analysing the proportion of positive partitions, using Poisson statistics (Dube et al., 2008; Quan et al., 2018; Whale et al., 2013). An important feature of sample partitioning is that it effectively concentrates the target sequences within isolated microreactors. This reduces competition between templates, making it possible to detect rare variants in a background of wild-type DNA and improves tolerance to inhibitors. End-point, binary detection makes dPCR particularly suitable for applications requiring high sensitivity and precise quantification of low-abundance targets (Quan et al., 2018). Commercial digital PCR systems use various partitioning strategies. The earliest developed method - chamber digital PCR (cdPCR), uses two-dimensional arrays of microchambers to split the reaction mixture. After thermal cycling, fluorescence imaging is used to identify positive reactions (Hindson et al., 2011; Ottesen et al., 2006; Quan et al., 2018).

Another approach, droplet digital PCR (ddPCR), uses oil emulsions to generate thousands of nanolitre droplets, which are thermocycled and subsequently analysed individually by fluorescence, in a process similar to flow cytometry (Baltrušis et al., 2018; Hindson et al., 2011). In studies conducted by Hindson et al. (2011), carefully optimised real-time PCR assays were shown to detect mutant alleles present at approximately 1%. However, when the same assays were applied using ddPCR, the mutant-to-wild-type ratio was improved by 20,000 times. More recently, nanoplate-based dPCR platforms have been developed. In these systems, the reaction mixture is evenly distributed across thousands of microwells within a sealed plate. Partitioning occurs entirely within this closed format, which minimises contamination risk and ensures uniform partition volumes, while also simplifying workflow. This design increases robustness, making nanoplate dPCR especially suitable for routine, high-throughput applications (Doğantürk et al., 2023).

### 5.3 Pyrosequencing

Among the most popular methods of detecting SNPs is pyrosequencing (Dolinská et al., 2023; Hinney et al., 2020; von Samson-Himmelstjerna et al., 2009). This is a real-time sequencing technique based on the release of inorganic pyrophosphate (PPi) upon the addition of a deoxyribonucleotide triphosphate to the end of a developing DNA strand. The sulfurylase reaction utilises pyrophosphate to release ATP, which is then used by luciferase to produce light. The amount of light generated during the reaction corresponds directly to the number of nucleotides that have been incorporated. Because each added nucleotide is known, the underlying DNA sequence can be read. Pyrosequencing is fast and straightforward, and it is particularly convenient for analysing multiple SNPs without requiring much extra time or effort (Harrington et al., 2013; Ronaghi, 2001; Royo et al., 2007; von Samson-Himmelstjerna et al., 2009; Yi-Ping et al., 2010).

### 5.4 Deep amplicon sequencing

In recent years, interest in deep amplicon sequencing, often combined with mixed amplicon metabarcoding for species identification (Avramenko et al., 2015), has grown significantly. The field of AR surveillance is currently undergoing a major transformation driven by the widespread availability of high-throughput massively parallel sequencing technologies (Antonopoulos et al., 2024a; Šlapeta et al., 2025). One of the major advantages of deep-amplicon sequencing is its suitability across multiple species and resistance markers (Antonopoulos et al., 2024a). In brief, targeted deep-amplicon sequencing for the detection of AR mutations involves the initial PCR amplification of the region of interest, in this case the *H. contortus* β-tubulin gene, followed by sequencing of the amplicons at depth using next-generation sequencing technology (Illumina being the platform of choice), alignment to the reference sequence, and estimation of the relative proportion of SNPs within the population (Avramenko et al., 2019). After being initially trialled in isolates from sheep farms in the United Kingdom (Avramenko et al., 2019), to date, targeted deep-amplicon sequencing of BZ resistance in *H. contortus* has been extensively demonstrated across the world in Australia for both sheep and goats (Francis and Šlapeta, 2023; Francis et al., 2024), in sheep in Canada (Queiroz et al., 2020), in goats in Mozambique (Guinda et al., 2025), in addition to being applied to a range of other animal and nematode species (Avramenko et al., 2020; Costa-Junior et al., 2021; Evans et al., 2021; Francis and Šlapeta, 2023; Kipp et al., 2025; Melville et al., 2020). This approach has also been used for the successful simultaneous detection of both BZ and LEV resistance markers in *H. contortus* in both sheep and goats in Australia (Francis et al., 2024).

### 5.5 Isothermal amplification

Isothermal amplification technologies, such as Loop-mediated Isothermal Amplification (LAMP) (Notomi et al., 2000) make use of a DNA polymerase with strand displacing activity (*Bst*), which eliminates the need for thermocycling as in conventional PCR reactions using *Taq* polymerase. This allows the reaction to be run at a constant temperature, in the case of LAMP 60-65^°^C (Notomi et al., 2000). LAMP reactions further make use of two essential (inner and outer) and one optional (loop) primer pairs, which bind to six distinct sites within the target region for polymerisation initiation (Nagamine et al., 2002; Notomi et al., 2000). Unlike PCR, LAMP amplicons form concatenated loop and dumbbell-shaped structures, with each amplicon yielding further additional sites for polymerisation initiation, which leads to the accumulation of 10^9^ copies per hour (Nagamine et al., 2002; Notomi et al., 2000). LAMP is, to date, the most widely used isothermal amplification technology (Martzy et al., 2019). A wide range of end-point detection methods are available, with varying suitability for detection in the field, and depending on whether quantification is required. For the detection of *H. contortus*, these include colourimetric and lateral flow detection (Khangembam et al., 2021), and fluorescent probe-based detection of single base changes (Antonopoulos et al., 2024b). LAMP has further been successfully adapted to detect BZ resistance SNPs. This was achieved by the development of allele-specific primers, which preferentially amplify when the SNP is present, being initially demonstrated for the detection of E198A in *H. contortus* populations in China (Tuersong et al., 2020). Subsequently, this was adapted to simultaneously detect the three principal SNPs in BZ resistance using synthetic DNA as the template with promising initial results, albeit with some limitations to overcome before use would be possible on field samples (Costa-Junior et al., 2022).

## 6 Prevalence of resistance-associated SNPs in different regions

To characterise how BZ resistance-associated polymorphisms in *H*. *contortus* are distributed across different countries, researchers have applied a range of molecular techniques and methodological approaches. Some studies have investigated the prevalence of resistant populations on farms by measuring the proportion of flocks that exhibit resistance alleles above a specified threshold, such as 10% or more (Ramünke et al., 2016). Other investigations have focused on estimating allele frequencies in pooled samples of parasites, offering a population-level perspective on the occurrence of SNPs, as detailed in Table 1. More detailed analyses, however, determined allele and genotype frequencies in individual larvae, allowing for the assessment of resistance allele fixation within populations (Table 2). All approaches targeted the β-tubulin isotype-1 gene, examining one or more of the key codons: 167, 198, and 200. Together, these datasets provide complementary insights into the spread and intensity of BZ resistance across regions.

**Table 1:** Frequencies of SNPs in isotype-1 β-tubulin of *H. contortus*, associated with benzimidazole resistance.

Country/Region	Year	Anim. spp	No. of pop.	No. of samp.	Method	F200Y	F167Y	E198A	Ref.
Sweden	2014-2019	sheep	67	174	droplet digital PCR	88.5% (14.1–100%)	0.74% (0–11%)	-	Baltrušis et al. (2020)
Austria (Styria, Salzburg)	2015	sheep	16	243	pyrosequencing	87-100%	-	0%	Hinney et al. (2020)
Brazil (Ceará State)	NA	sheep	20	Approx. 40 per pop.	qPCR	34.16% (±12.13%)	58.31% (±18.89%)	0.02–0.13%	Santos et al. (2017)
Brazil (Paraná)	NA	sheep and goats	18	669	qPCR	46.4-72%	15.7-23.8%	0%	do Prado et al. (2024)
Canada (Quebec)	2011,2012	sheep	8	20 per pop.	pyrosequencing	85.4%	6%	0%	Barrère et al., (2013
Poland	2017-2024	goats	81	NA	pyrosequencing	98% (91%–99%)	-	99% (93%-99%)	Nowek et al. (2026)

Year-year of sample collection; NA-data not available; Anim. spp-species of animals from which samples were collected; No. of pop. – number of populations; No. of samp. – number of samples; Method – molecular method applied; Ref. – reference, - - not investigated.

**Table 2:** Frequencies of isotype-1 β-tubulin SNPs and associated genotypes in individual *H. contortus* larvae.

Country/Region	Year	Anim. spp	No. of pop.	No. of samp.	Method	F200Y	F167Y	E198A	Ref.
Bosnia and Herzegovina	2021-2022	sheep	19	200	qPCR	RR: 77.8%, RS: 15.5%, SS: 6.7%	-	-	Kapo et al. (2024)
Bosnia and Herzegovina	2021-2022	goats	19	40	qPCR	RR: 100%	-	-	Kapo et al. (2024)
Greece	NA	sheep	53	55	allele- specific multiplex PCR	RR: 96.9%, RS: 3.1%, SS: 0%	-	-	Arsenopoulos et al. (2020)
Greece	NA	goats	55	63	allele-specific multiplex PCR	RR: 100%	-	-	Arsenopoulos et al. (2020)
Brazil (Ceará state)	NA	sheep	1	74	qPCR	26.24% RR: 6.88%	69.08% RR: 47.72%	0%	Araújo-Filho et al. (2021)

China (Hebei, Inner Mongolia, Liangning, Heilongjiang, Hubei, Yunnan, Shaanxi, Guangxi)	NA	sheep and goats	8	NA	PCR-sequencing	0-31%	0%	0-70%	Zhang et al. (2016)
China (Minfeng and Hejing)	NA	sheep	12	52	PCR-sequencing	13.33-14%	0%	30-40%	Tuerhong et al. (2024)
India (Tamil Nadu, Andhra Pradesh, Kerala and Karnataka)	2011	sheep, goats, cattle, buffalo	23	23	pyrosequencing	9-84%	0%	8-18%	Chaudhry et al. (2015)

Year-year of sample collection; NA-data not available; Anim. spp-species of animals from which samples were collected; No. of pop. – number of populations; No. of samp. – number of samples; Method – molecular method applied; Ref. – reference; **-** - not investigated.

RR-homozygous resistant genotype, RS- heterozygous resistant genotype, SS- susceptible genotype.

In Europe, the F200Y mutation has been consistently recognised as the major resistance-associated SNP, while F167Y and E198A typically occur at much lower frequencies. Data from Switzerland and Italy showed that the prevalence of *H. contortus* populations carrying resistant alleles changed noticeably over time (Ramünke et al., 2016). In north-eastern Switzerland, the proportion of sheep farms with a frequency of ≥10% of the F200Y mutation in *H. contortus* was very high, reaching 84.2% in 2012 and increasing to 96.7% in 2013. In contrast, in southwestern Italy, the corresponding prevalence was considerably lower, at 28.6% and 44.4% in 2012 and 2013, respectively. In both regions, populations exceeding the 10% threshold for F167Y and E198A were not detected; however, the 95% confidence intervals indicated that these SNPs could still occur at low prevalence levels, in Switzerland (0–16.8% in 2012 and 0–9.5% in 2013) and in Italy (0–35.4% in 2012 and 0–29.9% in 2013). It has been suggested that when at least 10% of individuals within a population carry a resistance genotype, the population can be considered BZ resistant (Barrère et al., 2013a).

Similar findings were reported in northern and central Europe, such as Sweden and Austria, where the F200Y mutation remained predominant, and other mutations occurred sporadically (Baltrušis et al., 2020; Hinney et al., 2020). Recent data from Norway also confirmed the widespread occurrence of BZ resistance in northern Europe. Screening of 169 sheep flocks across all major regions (2021–2022) revealed the F200Y mutation in 73% of larval samples from lambs and 69% from ewes. Although resistance was assessed at the flock level rather than by allele frequency within parasite populations, the findings indicate that BZ resistance is already broadly established in Norwegian sheep farms (Gravdal et al., 2023).

Comparable trends were observed beyond Europe. Studies conducted in Brazil (Ceará State) and Canada revealed that both F200Y and F167Y were detected at varying levels, reflecting regional differences in parasite populations (Barrère et al., 2013b; Santos et al., 2017) (Table 1). In these regions, F200Y generally remained the dominant mutation, while F167Y appeared more sporadically and E198A was rarely detected. Analyses performed at the individual larva level provided further insight into the genetic composition of resistant populations (Table 2). In south-eastern Europe, nearly all isolates from sheep and goats in Bosnia and Herzegovina and Greece carried the homozygous resistant genotype at codon 200 (Arsenopoulos et al., 2020; Kapo et al., 2024). Beyond Europe, resistance patterns were more variable: F167Y dominated in north-eastern Brazil (Ceará state) (Araújo-Filho et al., 2021), whereas F200Y was more common in southern regions (do Prado et al., 2024). Both F200Y and E198A were found in Chinese isolates, while F167Y was absent (Tuerhong et al., 2024; Zhang et al., 2016). In southern India, F200Y again represented the principal resistance marker, with E198A detected at lower frequencies (Chaudhry et al., 2015). Although the sample sizes in Chinese and Indian studies were relatively small, they still provide valuable insight into the independent evolution of resistance in distinct parasite populations under local selective pressures. Recently conducted study in Poland revealed high level of both F200Y and E198A in 81 goat herds, (Nowek et al., 2026), what correspond to data from prevalence studies on BZ resistance assessed by *in vivo* and *in vitro* methods (Mickiewicz et al., 2019, 2020, 2021). This is the first large-scale study providing data on the occurrence of SNPs in goat herds in Poland.

## 7 Discussion

As AR spreads among livestock GINs in European nations, it is necessary to track and detect its presence at a local level. Studies that explore mutations associated with BZ resistance in *H. contortus* allow for a more precise understanding of the emergence and spread of resistance compared to other drug classes and parasite species. Long-term and recurrent use of BZs, as well as underdosing, undoubtedly place significant selection pressure on nematode populations (Chartier et al., 2001; Knapp-Lawitzke et al., 2015). *H. contortus* rapidly develops AR, even after only a few years of exposure. This adaptability originates from the immense genetic variation found within its populations (Doyle et al., 2022). This variation is primarily due to their large population sizes, which enable effective selection (Gilleard and Redman, 2016; Prichard, 2001). Even though the F200Y mutation appears to be consistently present in *H. contortus* populations worldwide, significant regional differences exist in the prevalence of the F167Y and E198A mutations. A straightforward explanation for these observed patterns could be that both mutations are initially rare in a particular area but eventually spread extensively through animal movement (Chaudhry et al., 2015; Gilleard and Redman, 2016). Comparing the results across regions reveals that although the same key codons (200, 167, 198) are typically investigated, the prevalence patterns of SNPs vary substantially. These differences most likely arise from several contributing factors, including local drug-use practices, the intensity of anthelmintic treatments, climatic conditions that influence larval survival, and the degree of animal movement or trade between farms and regions (Araújo-Filho et al., 2021; Baltrušis et al., 2020; Charlier et al., 2022; Claerebout et al., 2020;Gilleard, 2013; Ramünke et al., 2016). Screening for resistance alleles serves as a critical tool in assessing the efficacy of anthelmintic treatments and implementing suitable control strategies. Given the interconnected nature of livestock trade and animal movement, coordinated international efforts are essential for effective AR monitoring and control. The spread of resistance alleles across regions emphasises the importance of coordinated surveillance programs and information sharing among countries, in addition to the critical importance of improved and standardisable molecular diagnostics (Antonopoulos et al., 2024a; Kaplan, 2020).

By promoting global cooperation and improving data exchange, we can respond more effectively to emerging resistance and help protect the long-term effectiveness of anthelmintic treatments. This collaborative approach also allows stakeholders to anticipate upcoming challenges and address them before they become more difficult to manage (Baltrušis et al., 2020; Gilleard and Redman, 2016; Knapp-Lawitzke et al., 2015; Learmount et al., 2016). Alongside environmental and management factors, differences in methodology should also be considered when interpreting regional patterns of SNP prevalence. The diagnostic performance of molecular tools, such as dPCR, pyrosequencing, or qPCR, can impact the detection of low-frequency resistance alleles. Compared to traditional *in vivo* and *in vitro* tests such as the faecal egg count reduction test (FECRT) or the egg hatch test (EHT), molecular techniques provide faster, more sensitive, and more reliable detection of BZ resistance (Avramenko et al., 2019; Baltrušis et al., 2020; Barrère et al., 2013b; Francis et al., 2024; Ramünke et al., 2016). Integrating molecular diagnostics into routine surveillance enables the detection of resistance earlier and with a more accurate assessment of resistance levels within parasite populations. Furthermore, sustainable control of *H. contortus* cannot rely solely on pharmacological approaches. Combining refugia-based management, strategic grazing, and biological control measures is essential for reducing selection pressure on parasite populations. These non-chemical strategies not only complement anthelmintic treatments but also help maintain their long-term effectiveness while supporting more sustainable and environmentally responsible parasite control (Hodgkinson et al., 2019; Hoste et al., 2015; van Wyk, 2001).

## 8 Conclusion

BZ resistance in *H. contortus* is a widespread and evolving challenge for small-ruminant production. While F200Y remains the predominant resistance-associated marker, marked regional variation in the occurrence of F167Y and E198A highlights the need for locally informed surveillance. In this context, the integration of molecular and genetic tools into routine resistance monitoring is no longer merely advantageous, but increasingly necessary, as it enables earlier detection of resistant populations, more accurate estimation of resistance allele frequencies, and more timely adaptation of treatment strategies. When integrated with routine molecular surveillance, the prudent use of anthelmintics and non-chemical control measures can support evidence-based parasite control, improve resistance management, and help preserve the long-term efficacy of available drugs.

## CRediT authorship contribution statement

**Zofia Nowek:** Conceptualization, Formal analysis, Investigation, Methodology, Writing – original draft. **Marcin Mickiewicz:** Conceptualization, Writing – original draft, Writing – review & editing. **Alistair Antonopoulos:** Conceptualization, Data curation, Writing – original draft, Writing – review & editing. **Adrian-Valentin Potărniche:** Writing – review & editing. **Kinga Biernacka:** Methodology, Writing – review & editing. **Rareş-Constantin Bejenariu:** Writing – review & editing. **Karolina Radziejewska:** Writing – review & editing. **Aija Mālniece:** Formal analysis, Writing – review & editing. **Jarosław Kaba:** Conceptualization, Supervision, Validation, Writing – original draft, Writing – review & editing.

## Declaration of competing interest

The authors declare that they have no known competing financial interests or personal relationships that could have appeared to influence the work reported in this paper.
